# The agreement between bronchoalveolar lavage, bronchial wash and sputum culture: a retrospective study

**DOI:** 10.1007/s15010-024-02238-5

**Published:** 2024-04-08

**Authors:** Anne-Eva Post, Erik Bathoorn, Douwe F. Postma, Dirk-Jan Slebos, Onno W. Akkerman

**Affiliations:** 1 Department of Pulmonary Diseases and Tuberculosis, University Medical Center Groningen, University of Groningen, Groningen, The Netherlands; 2 Department of Medical Microbiology, University Medical Center Groningen, University of Groningen, Groningen, The Netherlands; 3 Department of Internal Medicine and Infectious Diseases, University Medical Center Groningen, University of Groningen, Groningen, The Netherlands

**Keywords:** Bronchoalveolar lavage, Bronchial wash, Sputum, Pneumonia

## Abstract

**Purpose:**

Bronchoalveolar lavage is commonly used in clinical practice for unresolved pneumonia. However, bronchoalveolar lavage is not suitable for all patients as it is an invasive procedure and can worsen oxygenation. The diagnostic value of bronchial wash and sputum has been debated extensively over the years. In this study, we aim to compare the diagnostic value in several pathogens of bronchoalveolar lavage and bronchial wash, and secondarily bronchoalveolar lavage and sputum.

**Methods:**

We retrospectively included all adult patients in our hospital who underwent bronchoalveolar lavage, bronchial wash, and where sputum sampling was done between January 1st of 2018 and December 31st of 2021. The intraclass correlation coefficient was computed for the three tests.

**Results:**

In total, 308 patients were included. We found a level of correlation of 0.819 and 0.865, respectively, between bronchoalveolar lavage and bronchial wash for two pathogens: *Staphylococcus aureus* and *Pseudomonas aeruginosa.* For *Stenotrophomonas maltophilia* and *Aspergillus fumigatus*, we found an intraclass correlation coefficient of 0.568 and 0.624, respectively. Between bronchoalveolar lavage and sputum, we found varying levels of agreement.

**Conclusion:**

Our study shows reasonably well agreement levels between bronchoalveolar lavage and bronchial wash, suggesting that bronchial wash could potentially be an alternative to bronchoalveolar lavage.

## Introduction

Pneumonia is a leading cause of morbidity and mortality in all age groups [1]. It remains a clinical diagnosis primarily, based on clinical signs and symptoms, laboratory investigation, and imaging. The gold standard to detect acute infections of the pulmonary parenchyma would still be a lung biopsy or autopsy, both of which are not wishful procedures in clinical practice.

Identifying the microbial etiology in pneumonia remains a challenge, as it is often difficult to collect a good quality specimen from the lower respiratory tract and blood cultures tend to remain negative [2]. Although, over the years, several methods to obtain specimens from the respiratory system have been designed, the causative pathogen is only defined in ~ 60–70% of etiological studies of pneumonia cases, whereas the yield in clinical practice is much lower (~ 30%) [2, 3]. Establishing the causative pathogen early in pneumonia is cardinal for reducing diagnostic delay and ensuring the administration of effective antibiotics, especially in an era of growing antibiotic resistance and rising health care costs [4].

Since the early 1900s culture of sputum is used to identify the pathogen in pneumonia [2].

Although it is the first step in in-hospital analysis, its sensitivity, reliability and impact on treatment decisions within various pathogens have been debated [4–6]. In addition to being difficult to obtain in subsets of patients and time consuming, sputum specimens are frequently contaminated with upper respiratory tract micro-organisms, leading to inconclusive results [7, 8]. Furthermore, empirical antibiotic use can often lead to false-negative culture results [9].

The increase of pneumonia in immunocompromised patients following the rise of AIDS diagnoses and organ transplantations in the 1970s asked for a distal diagnostic sampling approach, as most of the opportunistic infections were not primarily located in the proximal bronchi [10]. Fiber-optic bronchoscopy and in particular bronchoalveolar lavage (BAL), in which a bronchoscope is used to instill saline in the bronchioles and alveoli to obtain a more distal specimen, became an option in diagnosing pathogens that do not normally colonize the upper respiratory tract [2, 10, 11].

Nowadays, BAL still plays an important role in the diagnosis of pneumonia in immunocompromised patients and in patients in which the probability of changing the initially empiric antibiotic regime is high [9, 12]. Although BAL is presented as a safe procedure with no absolute contraindications, its invasive nature and variable diagnostic values makes it not routinely performed on patients [13]. Nevertheless, the latest clinical practice guidelines of the Infectious Disease Society of America (IDSA) recommends lower respiratory tract specimens for community acquired pneumonia (CAP) patients who are immunocompromised or in which treatment fails and for all patients with a suspicion of hospital acquired- or ventilator-associated pneumonia (HAP/VAP) [4, 13].

Bronchial wash (BW), where in contrast to in BAL, the bronchoscope does not isolate the bronchiole of the rest of the airways, is a method used solely for specimens of the major airways not representing the bronchioles and alveoli. Contamination by the upper airways is inevitable. Therefore, this method has been regarded as useless in the diagnosis of not strictly pathogenic microbes and results on associated risks and contraindications compared to BAL have not been documented [10].

In 2020, the International Society for Heart and Lung Transplantation (ISHLT) conducted a survey on BAL and BW definition and performance with 114 lung transplant centers across the world. This survey showed a considerable diversity in BAL and BW performance and techniques worldwide. Although guidelines on performance of BAL have been published in the past years, guidance on the BW technique was not provided [14]. This implies a less clear cut distinction between the two procedures in clinical practice, which challenges the frequent use of BAL.

The lack of clarity around the diagnostic value of BW leads to the frequent use of BAL despite the disadvantages for certain patient groups. This study aims to compare the diagnostic agreement between culture and PCR results of BAL, BW and sputum of the most common pathogens causing pneumonia, in order to explore the value of less invasive alternative diagnostic methods for pneumonia.

## Methods

### Setting

This retrospective study was conducted at the University Medical Center of Groningen (UMCG) in the Netherlands. All patients who underwent BAL as well as BW during admission or an outpatient department visit between January 1st, 2018 and December 31st, 2021 were included. An extra cohort was defined by patients who additionally to BAL and BW specimens had a sputum specimen taken during the evaluation.

### Study design

Retrospectively, we included adults (≥ 18 years old) who underwent BAL as well as BW as those were considered clinically indicated by the individual physician. Patients of whom in addition sputum specimens were taken during evaluation, were additionally added to a subset.

The first BAL and BW results of an evaluation of a patient were included. In patients of whom multiple evaluations were done during the 3 years, only the first analysis was included. Sputum results were included within a period of 3 days before and 3 days after BAL and BW performance. BAL and BW specimens were always taken on the same day.

Microbiological results of the specimens were obtained from the database of the department of microbiology of the hospital.

Analyses were selectively performed on predetermined micro-organisms, specifically selected for their clinical relevance (Table 1).

**Table 1:** Analyzed micro-organisms

Fungal micro-organisms
*Aspergillus fumigatus*
*Pneumocystis jirovecii*
Bacterial micro-organisms
*Staphylococcus aureus*
*Pseudomonas aeruginosa*
*Stenotrophomonas maltophilia*
*Mycobacterium tuberculosis*
*Non-tuberculous mycobacterium (not specified)*

### Review of clinical records

Patient demographics (gender, age, smoking status) and clinical characteristics (white blood cell count, CRP, comorbidities, immunosuppressive status, radiological signs, pre-existing medication use, department of evaluation) were obtained from the electronic medical record system. Additionally, we collected details about pre-existent use of antibiotics and antimycotics.

The CRP and white blood cell count closest to the execution of the BAL and BW were obtained from the medical records with a maximum of 3 days before or after the procedure. The radiologic imaginary closest in time to the BAL were extracted from the files with a maximum of 5 days before or after the procedure.

### Sampling techniques

For BAL, 150–200 ml isotonic saline was instilled in the periphery of the airways and the lung parenchyma of the affected area, while positioning the bronchoscope in wedge position. The BAL was performed as appropriate where the radiologic abnormality was localized. In patients with diffuse radiologic infiltrates the BAL was performed in the middle lobe or lingula of the affected lung. In BW, a bronchoscope was used to instil 20–40 ml isotonic saline into a lobar or segmental airway after which it was aspirated. In this study, BW was principally performed before BAL in the same bronchoscopic procedure to prevent contamination, however, this was documented systematically in medical records, nor was a protected catheter employed. Sputum specimens were spontaneously expectorated into sterile containers by the patients [15, 16].

### Microbiology

Aliquots of fluid specimens obtained from BAL or BW were plated on media to culture aerobic bacteria, anaerobic bacteria, fungi and mycobacteria by standard protocols in routine diagnostics. Sputum specimens were plated on blood agar, chocolate agar and Mackonckey. For Aspergillus species, an aspergillus DNA PCR was performed per in-house protocol. A Giemsa stain is used for *Pneumocystis jirovecii* detection in combination with a PCR test. Tuberculous as well as non-tuberculous mycobacteria were detected using an auramine staining, additionally an in-house PCR was done on mycobacterium species and *Mycobacterium tuberculosis*. Bacterial culture was considered positive when there was an excess of or a pure culture of one specific species or when leukocytes as well as respiratory pathogens were found in the preparation.

### Definitions

#### Pneumonia

Pneumonia was defined by clinical findings consisting of a fever, new onset of cough (or deterioration of existing cough) or dyspnea, next to a new consolidation on radiological findings (by chest X-ray or chest CT).

Pneumonias are categorized in community acquired pneumonia (CAP), hospital acquired pneumonia (HAP), and ventilator associated pneumonia (VAP), depending on the site of acquisition.

#### Immunocompromised state

Immunosuppression is defined as the use of high dose steroids (use of prednisone 40 mg per day or its equivalent, for more than 2 weeks), the use of chemotherapy, diagnosis of leukemia, lymphoma or HIV with a CD4 count < 400, history of organ or stem cell transplantation with the administration of immunosuppressants, neutropenic patients (absolute neutrophil count < 500/mm^3^) or a history of splenectomy [17].

### Statistical analysis

BAL was considered the golden standard for comparison. For numeric values, medians and interquartile ranges are reported. Categorical values are reported as absolute numbers and percentages (n, %). All statistical analyses were performed using SPSS version 28 (IBM, SPSS, Chicago, IL).

The intra-class correlation coefficient (ICC) was used to quantify the degree of agreement between the different methods. An ICC of 0 indicating no agreement, 0–0.20 as poor agreement, 0.20–0.41 as fair agreement, 0.41–0.61 as moderate agreement, 0.61–0.81 as substantial agreement, 0.81–0.99 as near perfect agreement and 1 as perfect agreement. A statistical difference was considered insignificant if *p* < 0.05.

## Results

**Fig. 1:**
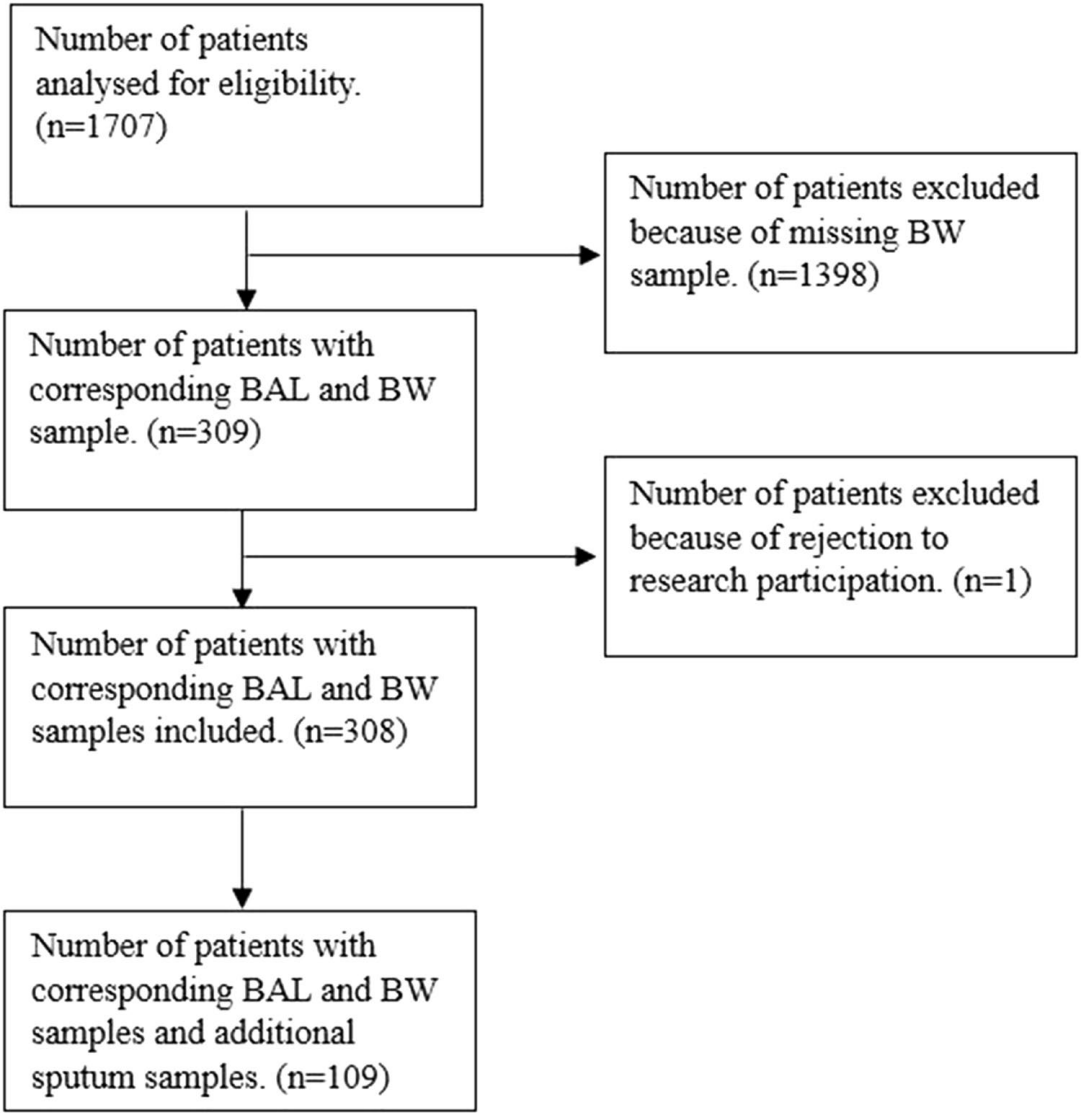
Flowchart of patient selection in the present study

A total of 1707 patients underwent BAL sampling in our centre. Only 309 patients underwent BAL and BW sampling on the same day. Subsequently, we excluded one patient because of a registration for objection against research participation. The remaining 308 patients were included and analysed. Additionally, in 109 of these patients, a sputum specimen was taken before or after BAL (Fig. 1).

The median age was 63 years (interquartile range [IQR] 55–69). 59 patients (19.2%) were evaluated on an outpatient basis and 70 patients (22.7%) were admitted to the intensive care unit. 131 patients (42.5%) were being treated for ongoing haematological malignancies. A further 218 of patients were immunocompromised during evaluation. Pulmonary comorbidities were present in 64 patients (20.7%): 30 (9.7%) with COPD, and 15 + 6 + 7 + 6 = 34 (11%) with other chronic respiratory diseases (Table 2).

**Table 2:** Baseline characteristics

Characteristics	Patients *N* = 308
Age, years	63 (55–69)
Female/male	102 (33.1)/206(66.9)
History of smoking	
Smoker	23 (7.5)
Never smoker	143 (46.4)
Former smoker	120 (39.0)
Unknown	22 (7.1)
CRP^a^	111 (54–200.5)
Leukocyte count	7.1 (2.2–11.5)
Outpatient/inpatient evaluations	59 (19.2)/ 249 (80.8)
Intensive care admission	70 (22.7)
Antibiotic administration prior to BAL^b^	207 (67.2)
Antimycotic administration prior to BAL	80 (26.0)
Comorbidities at time of sampling	300 (97.4)
Hematological malignancies	131 (42.5)
Post-transplantation	59 (19.2)
COPD^c^	30 (9.7)
Astma	15 (4.9)
Cystic fibrosis	6 (1.9)
Interstitial pulmonary disorders	7 (2.3)
Bronciectasis	6 (1.9)
HIV/AIDS^d^	3 (1.0)
COVID-19	25 (8.1)
Immunocompromised state	218 (70.8)

Values are presented as *N* (%) or median (25th–75th percentile), COPD chronic obstructive pulmonary disease

^a^C-reactive protein

^b^Bronchoalveolar lavage

^c^Chronic obstructive pulmonary disease

^d^Human papilloma virus/Acquired immunodeficiency syndrome

### Microbiological results

The main results of identified pathogens in the BAL fluid (BALF) are highlighted here. A more detailed portrayal of findings is given in Table 3.

**Table 3:** Microbiological findings and contributions of different methods to diagnostic yield

Micro-organism	Total No BAL Results^a^	Positive findings in BAL^b^	Total No. BW Results	Positive findings in BW	Total No. Sputum Results	Positive findings In sputum
*Staphylococcus aureus*	307	14	304	15	109	12
*Immunocompromised*	218	6	216	6	72	5
*Immunocompetent*	89	8	88	9	37	7
*Pseudomonas aeruginosa*	307	11	304	12	109	6
*Immunocompromised*	218	9	216	10	72	4
*Immunocompetent*	89	2	88	2	37	2
*Stenotrophomonas maltophilia*	307	2	304	5	109	2
*Immunocompromised*	218	1	216	2	72	1
*Immunocompetent*	89	1	88	3	37	1
*Aspergillus fumigatus*	306	22	304	24	68	3
*Immunocompromised*	217	10	215	15	49	0
*Immunocompetent*	89	12	89	9	19	3
*Mycobacterium tuberculosis*	216	1	115	1	6	0
*Immunocompromised*	164	0	79	0	4	0
*Immunocompetent*	52	1	36	1	2	0
*Pneumocystis jirovecii*	188	37	2	0	1	0
*Immunocompromised*	158	34	2	0	1	0
*Immunocompetent*	30	3	0	0	0	0
*Non-tuberculous mycobacteria*	220	1	100	2	8	0
*Immunocompromised*	164	1	69	1	4	0
*Immunocompetent*	56	0	31	1	4	0

^a^Total number of specimens plated for specific micro-organism

^b^Total number of positive findings in specific diagnostic specimen for micro-organism

#### BAL findings

In the standard bacterial culture performed on 307 BALF, *Staphylococcus aureus* was found most frequently (*n* = 14, 4.6%), followed by *Pseudomonas aeruginosa* (*n* = 11, 3.6%). *Stenotrophomonas maltophilia* was only found twice in the BALF analyzed (0.7%).

*Aspergillus* PCR testing was performed on 306 of those specimens, in which *Aspergillus Fumigatus* was found 22 times (7.2%). Another fungal pathogen, *Pneumocystic jirovecii* was found 37 times in the 188 specimens analyzed (19.7%).

Both *Mycobacterium tuberculosis* (MTB) and *non-tuberculous mycobacteria* (NTM) were found only once in the BALF analyzed.

#### BW findings

In BW, a general culture and *Aspergillus* DNA PCR were performed on 304 BW. *Staphylococcus aureus* was found in 15 specimens followed by *Pseudomonas aeruginosa* in 12 specimens (4.9%; 3.9%). *Stenotrophomonas maltophilia* was seen 5 times in the BW (1.6%). *Aspergillus Fumigatus* was found in 24 of the 304 specimens (7.9%).

*Pneumocystic jirovecii* analysis was only performed on two BW specimens, no positive *Pneumocystic jirovecii* results were found in these specimens (0.0%). Analyses for MTB and NTM were also performed on less BW specimens, 115 and 100 times, respectively.

#### Sputum findings

In general, sputum cultures performed on 109 sputum specimens, *Staphylococcus aureus* was found in 12 specimens, *Pseudomonas aeruginosa* in six specimens and *Stenotrophomonas maltophilia* in two specimens (11.0%; 5.5%; 1.8%). *Aspergillus fumigatus* was found in three sputum specimens, *Pneumocystic jirovecii* testing was only performed once and resulted in a negative test (4.4%; 0.0%). MTB and NTM was only tested for among a few of sputum specimens, this led to negative results in all those specimens.

### Comparing diagnostic efficacy

#### BALF versus BW

We found 304 matched pairs of BALF and BW specimens in our study group. For four micro-organisms, the ICC was calculated.

For *Staphylococcus aureus*, the ICC value between BALF and BW was 0.819. That portrays a near perfect agreement between the two tests in detecting *Staphylococcus aureus*. Additionally, a near perfect agreement was also found in the ICC value for *Pseudomonas aeruginosa*, that showed an ICC of 0.865.

A moderate agreement between the two tests was found for *Stenotrophomonas maltophilia*, showing a slightly lower ICC value of 0.568.

For *Aspergillus fumigatus*, the consistency between the two tests was substantial (ICC = 0.624).

#### BALF vs. Sputum

108 pairs of BALF and sputum specimens were collected. The measure of agreement between the two diagnostic methods obtained for *Staphylococcus aureus* was fairly good (ICC = 0.402). For Pseudomonas aeruginosa, a substantial agreement was found (ICC = 0.792). A perfect agreement was found in our study group for *Stenotrophomonas maltophilia* (ICC = 1.000*)*. For *Aspergillus fumigatus*, the ICC value was 0.413, showing a fair agreement between the two tests.

## Discussion

This study aimed to compare the diagnostic value of BAL and BW and secondarily, BAL and sputum specimens in: *Staphylococcus aureus*, *Pseudomonas aeruginosa*, *Stenotrophomonas maltophilia* and *Aspergillus fumigatus*. A near perfect agreement level between BAL and BW for *Staphylococcus aureus* and *Pseudomonas aeruginosa* was found. Secondarily, comparing BAL and sputum specimens varying levels of agreement between the two methods were found for the four microorganisms.

Firstly, our results suggest that BAL and BW have a strong agreement in identifying the presence or absence of *Staphylococcus aureus* and *Pseudomonas aeruginosa.* Although BAL is the leading gold standard in unresolved, high-risk CAP and all HAP/VAP patients, there have been questions about its sensitivity and specificity as well as suitability in all patients over the past 30 years [4, 13]. Limited information on the subject is available, since many studies did not analyze BAL and BW separately [18].

In 2002, Pinckard et al. analyzed the additional diagnostic value of BW next to BAL. They recommended active questioning of the need for BW, because of its often ambiguous results without clinical consequences [19]. After this study, the diagnostic value of BW has only been studied a couple of times in different clinical situations. Kim et al. studied the value of BW in sputum scarce or smear negative with suspected MTB infection. BAL showed higher diagnostic value in this group [20].

A small study in 32 immunocompromised patients on mechanical ventilation in an ICU showed comparable correlations as our study between mini-BAL and BAL. Although their mini-BAL procedure was comparable in terms of volume and sampling technique as our BW, it was more controlled to prevent contamination. They used a separate protected catheter for their mini-BAL to obtain a volume of 20 ml saline solution instilled into the airways, and performed a normal lavage with a full flexible bronchoscope after. [21]

Sputum specimens to identify the cause of pneumonia are nowadays only indicated for CAP in which the chances of changing antibiotic management is substantial and in patients who are at high risk for clinical deterioration [4].

In 2002, Ewig et al. found that there was no contribution of sputum cultures to in-hospital pneumonia management since its limited diagnostic efficacy [5]. Since then various studies are done in various settings not reaching a consensus about the diagnostic efficacy of sputum specimens [4]. Zhang et al. is one of the first studies comparing the agreement between BAL and sputum culture. They found a moderate agreement between sputum culture and BAL culture in their cohort of children admitted to the hospital with suspected CAP. In previous other studies, the comparison between BAL and sputum culture was made but their agreement was not analyzed [6].

In HAP, the comparison between BAL and sputum culture has scarcely been studied. A lot of the knowledge about sputum culture in HAP is extrapolated from studies about VAP or CAP. The recent IDSA guidelines reviewed three studies showing comparable diagnostic yields in VAP for non-invasive and invasive techniques, although these had their methodological limitations. Evidence for comparable diagnostic yields in HAP is not presented. Nevertheless, the guideline suggests non-invasive sampling in HAP patients, invasive sampling is recommended when the individual clinician sees it fitting [13].

Our results showing varying agreement between sputum and BAL among the four micro-organisms seems to be in line with previous knowledge. Especially, when noting the fact that in our study group, the division between CAP, HAP and VAP patients was not made.

This study has certain limitations. A first limitation of our study is the single-centered nature, which restricts the generalizability of the results to different health care settings. Additionally, retrospective inclusion made it unfeasible to ascertain the precise methodologies employed during the BAL and BW procedures, which may have resulted in diminished accuracy of the study outcomes. Moreover, during the execution of the procedures, BW was often performed in the same bronchoscopic procedure as BAL. During these procedures, methods to prevent contamination of the instilled saline were not employed. This may have influenced the results of this study. Furthermore, it was impossible to completely avoid selection bias as the decision to perform BAL and BW, and the microbiological determinations were made by the individual clinician. Besides, although used in many studies on the subject, BAL is not a perfect golden standard, limiting this study on providing results on diagnostic yield and specific diagnostic values. However, we believe an imperfect golden standard is still useful for comparison in diagnostic value in the same groups of patients.

Another finding limiting our study is the relatively small sample size for the specific pathogens. Although the scarcity of Enterobacteriaceae found in HAP/VAP patients in the ICU in this study can likely be attributed to the administration of selective digestive decontamination. Nevertheless, to the best of our knowledge, this is one of the studies with the largest sample size on the subject in the last 10 years and the first differentiating between diagnostic values for specific pathogens.

Another strength of this study is that we performed an individual chart review for every patient yielding us a lot of additional information about the patients included. Besides, the interval between cultures is considered in this study, which has been overlooked in other studies. In this study, bronchoscopic specimens were collected on the same day and sputum specimens were collected within a 3-day period around the day of BAL, this way avoiding a great amount of long interval bias.

Additionally, this study contributes to a need highlighted by the Infectious Disease Society of America for knowledge about the diagnostic methods and tools for identifying HAP/VAP in 2017 [13].

The results of this study suggest a comparable diagnostic value between BAL and BW in clinically relevant bacterial pathogens. However, we assume that this can be partly attributed to the fact that execution of BAL and BW is variable between physicians and that these procedures are, subsequently, not as distinctive as defined. Additionally, contamination between BAL and BW may have influenced the results. Future studies should portray results on cohorts in which these procedures have been executed strictly according to protocol. Likewise, new studies should focus on specific subgroups and portray a broader description of the spectrum of pathogens. The agreement between sputum and BAL is variable, depending on the specific pathogen in our patient group, as it is described in previous studies on the subject. Additional studies should determine if sputum specimens can have a possible role in diagnosing infectious pneumonia in some pathogens.

## Data Availability

The data that support the findings of this study are available from the corresponding author, OWA, upon reasonable request.
